# Dental Therapeutics

**Published:** 1873-09

**Authors:** G. W Gray


### DENTAL THERAPEUTICS.

*Read before the Oregon State Dental Society, June 25th* 1873, *by G. W. Gray, D. D. S.*

*Mr. President, Gentlemen and Members of the Oregon State Dental Society:*

There are many subjects of interest that might very profitably claim our attention, but none of them more deserving of careful study and earnest investigation, than the one very recently assigned me to write upon. In treating upon this subject, I wish first to refer you back to the time when God created the heavens and the earth, the sea and all things therein in which is the breath of life, "and God said Let the earth bring forth the living creature *after his kind* ; so God created man in *his own image*, in the image of God created he him ; male and female created he them ; and God said unto them, Be fruitful, and multiply. and replenish the earth." Man being created in the image and likeness of God was undoubtedly a perfect man in all his parts, anatomically and physiologically speaking, and did not spring from the ape, and the ape from the tidal animal, and the tidal animal from some ultimate monad, in whom all vital functions are reduced to the merest rudiments (according to Darwinism). But when Adam and Eve were created and placed in the Garden of Eden, they were created a full-sized man and woman, with beautiful arches of perfectly developed permanent sets of teeth. (They probably being the only persons who ever had the permanent teeth without first having a temporary set.) Those first given them having undoubtly been intended by their Creator to last them, not only for three score years and ten, but during their natural lives—or for hundreds of years.

Had man continued to comply with the mandates of his divine Creator by bringing forth *after his kind* untill this, what would have been the result ? Alas ! gentlemen, where would have been your calling and mine ? I fear we would have been compelled to seek other fields of usefulness, in which to cultivate and employ our talents. But the teeth of the present generation of mankind are not so perfect ; and do not appear " like a flock of sheep that are evenly shorn which came up from the washing," and the mouths most sweet and altogether lovely ; but disease and death are abroad in the land, making serious inroads among us and cutting off not only its hundreds and thousands, but millions of teeth annually. You may notice the infant of tender years suffering untold miseries by decayed and aching teeth, and in many cases called to an early and permature grave. But should its power of endurance prove sufficient, and were it to succeed in enduring all the pain occurring during the various stages of cutting and shedding the deciduous set, the permanent teeth taking their place one by one, its troubles are but begun ; for disease continues its destructive effects upon the permanent, as well as the temporary set, and to preserve them requires the most skillful operations, that can be preformed by the scientific dentist; and even then they frequently decay away, are finally removed and replaced by artificial teeth—often at a very early period in life—or, as is sometimes the case, the permanent teeth never make their appearance in the jaw. I read of a case recently, where a dentist made a set of artificial teeth for a lady who never had any natural teeth; and I have a case in my own practice, of a gentleman for whom, some years since, I inserted a partial set of artificial teeth, the natural ones never having made their appearance.

In reflecting upon the teeth and their associate parts, in their present state of retrogradation, it certainly becomes us to study diligently those agencies, or that branch of medicine which relates to the treatment of their disease—that is, dental therapeutics—and our first inquiry is, what is dental therapeutics? "Dental," pertaining to the teeth; "therapeutics" is curative; that pertains to the healing art; that is concerned in discovering and applying remedies for disease; or that part of medicine which respects the discovery and application of remedies for disease, or teaches the use of diet and of medicines. As to how substances not used for food were first discovered and began to be employed as medicines, is very uncertain—whether man himself instinctively resorted to them, or only in imitation of the lower animals, ate certain plants when suffering pain from various sources. The art of using them is usually thought to be a divine gift to man. Undoubtedly, the instinctive cravings which often manifest themselves in desire, must have led the way to an experimental use of many substances resembling, in their physical qualities, those things which were the objects of desire; and thus, man was led by his rational faculties to make experiments, compare and analyze the results of his observations—experience being really the only true ground upon which curative effects could be expected from medicine. All the positive knowledge we now possess concerning medicine, is the result of accumulated observations of their effects, in many cases, during hundreds, and even thousands of years. Medicine can only be regarded as the handmaid of nature—

the really active and efficient agent in the restoring of health, and can do no more than remove the impediments in nature's path, support her when weak, restrain and guide her when she is inclined to stray. In studying the relation of medicine to the cure of disease, it is necessary to take into consideration two propositions : first, does the medicine really produce a cure ; second, in what manner does it operate. The former of these questions can only be answered by the results of experience—the latter may be solved by an analysis of the same experience, so as to detect the different steps by which the curative result is obtained. The avenues by which medicines are introduced into the system for the cure of disease are, either by external application, injection into the veins, or by introducing them into the stomach ; the latter mode being the best adapted by nature to accomplish the end desired, and in most cases is the one usually employed : but in treating diseases of the dental organs, local applications are perhaps more generally depended upon. There are various influences and deviations present in patients, which must have their due consideration in the application of remedial agents for the cure of disease ; such as race, climate, season, age, sex, habit, idiosyncrasies, mental emotions, etc., etc.; and to use medicines correctly, an accurate knowledge should be possessed concerning the diseases they are employed to cure.

But it is not the design of this paper to enter into a lengthy discussion or description of the various diseases of the dental organs, and the strong sympathy existing between those organs and other parts of the system, and how to treat them in all their varied and complicated forms ; but simply hint at what might be done towards preventing those diseases from occurring. The old adage says, "an ounce of prevention is better than a pound of cure;" and it is a generally acknowledged fact, that the teeth, like other parts of the body, usually resemble those of the parents ; and if those of the father or mother are defective or irregular, a similar imperfection can usually be found in those of the offspring. "Like begets like," and "as the twig is bent the tree is inclined." So if an improvement is made in the teeth of coming generations, it must be accomplished through the offspring, by commencing with the early growth of tooth life, and adopting a properly-chosen and well-directed course of dietetics, supplying those necessary bone-making materials through the medium of the circulation; first to the mother, during utero-gestation, and the nursing period of child life, then to the child, during all the formative stages of tooth substance. The engineer cannot get up power to propel his machinery without a continuous supply of wood, water and fire, the necessary elements to be used in generating steam (the propelling power). Neither can the teeth be made good or kept in a perfect state of preservation, unless they are constantly supplied with those bony ingredients that enter so largely into their composition. It is truly gratifying to know that the profession are waking up to the importance of this subject, and are making an effort towards reformation; and I hope instead of the dental practitioner devoting all his leisure moments to the invention or construction of improved dental instruments, burring engines, patent plates, teeth, etc., etc., for makiog artificial dentures, at least a portion of that time may be set aside and devoted to the studying out and discovering of some means by which nature may be enabled to work out her own reform, and eventually get back to her primitive state of perfection and beauty.

